# Regulatory T Cells Are Dispensable for Tolerance to RBC Antigens

**DOI:** 10.3389/fimmu.2016.00348

**Published:** 2016-09-19

**Authors:** Amanda L. Richards, Linda M. Kapp, Xiaohong Wang, Heather L. Howie, Krystalyn E. Hudson

**Affiliations:** ^1^Bloodworks Northwest Research Institute, Seattle, WA, USA

**Keywords:** tolerance, autoimmunity, autoimmune hemolytic anemia, regulatory T cells, red blood cell, immunological tolerance

## Abstract

Autoimmune hemolytic anemia (AIHA) occurs when pathogenic autoantibodies against red blood cell (RBC) antigens are generated. While the basic disease pathology of AIHA is well studied, the underlying mechanism(s) behind the failure in tolerance to RBC autoantigens are poorly understood. Thus, to investigate the tolerance mechanisms required for the establishment and maintenance of tolerance to RBC antigens, we developed a novel murine model. With this model, we evaluated the role of regulatory T cells (Tregs) in tolerance to RBC-specific antigens. Herein, we show that neither sustained depletion of Tregs nor immunization with RBC-specific proteins in conjunction with Treg depletion led to RBC-specific autoantibody generation. Thus, these studies demonstrate that Tregs are not required to prevent autoantibodies to RBCs and suggest that other tolerance mechanisms are likely involved.

## Introduction

Loss of humoral tolerance to red blood cell (RBC) antigens may lead to development of pathogenic autoantibodies and result in autoimmune hemolytic anemia (AIHA) (1). When this occurs, it can have devastating effects. Immunosuppression can be efficacious, but many patients relapse within 1-year post-treatment (2–4). Transfusion support of AIHA patients can be challenging, as many RBC autoantigens are common to essentially all RBC donors; thus, transfusable units may be limited, and in some cases, no compatible RBCs are available to transfuse. While the clinical presentation of AIHA has been well described, the basic pathogenesis of primary AIHA remains poorly understood (5).

Tolerance to autoantigens is achieved through a coordinated effort between central and peripheral tolerance mechanisms (6–8). Central tolerance is the education of developing B and T lymphocytes to autoantigens; self-reactive lymphocytes undergo deletion, receptor editing, anergy, or persist in a regulatory capacity. Although most self-reactive cells are eliminated centrally, some autoreactive lymphocytes escape tolerance and persist and then mature in the periphery (9–11). However, unwanted activation of peripheral autoreactive cells is typically prevented by peripheral tolerance mechanisms, including regulatory cells, immunosuppressive cytokines, and antigen-presenting cells with tolerizing phenotypes (12, 13).

Despite multiple checkpoints to ensure tolerance to self-antigens, worldwide prevalence of autoimmunity is ~12%, indicating tolerance mechanisms break down (14). Moreover, RBC-specific autoantibodies are detectable in 0.1% of asymptomatic blood donors, suggesting that tolerance to RBC autoantigens frequently fail. Thus, to elucidate mechanisms of tolerance to RBC antigens and identify which tolerance pathways fail thereby leading to autoimmunity, we have developed a model of RBC autoimmunity using the HEL–OVA–Duffy (HOD) mouse. The HOD mouse expresses a triple fusion protein consisting of hen egg lysozyme (HEL), ovalbumin (OVA), and Duffy (HEL–OVA–Duffy) expressed behind an RBC-specific promoter (15). The HOD antigen is detected on RBC precursors and is expressed at levels comparable to naturally occurring RBC antigens (16, 17). Using the HOD model, we previously reported that HOD mice are profoundly tolerant to both HOD RBCs, and also HEL and OVA protein-based immunizations. In mice expressing the HOD transgene, autoreactive HOD-specific T cells are detectable in the periphery, but are non-functional, as indicated by lack of T cell proliferation or activation upon stimulation with cognate antigen. However, HOD-reactive T cells function normally if they develop in the absence of the HOD antigen (e.g., wild-type mice). Unlike T cells, HOD autoreactive B cells survive central and peripheral tolerance in HOD transgenic mice and are fully capable of maturing into autoantibody-secreting plasma cells after receipt of functional autoreactive HOD-reactive T cells (through adoptive transfer). Thus, the HOD system identifies T cell anergy, or non-responsiveness, as a critical checkpoint in the prevention of AIHA.

Regulatory T cells (Tregs) play a major role in tolerance to self-antigens, as congenital absence or transient depletion of Tregs has been correlated with early onset autoimmunity (18). A principle characteristic of Tregs is that they are unresponsive to T cell receptor stimulation and they can render other T cells anergic through immunosuppressive cytokine secretion or alteration of the availability of IL-2, a cytokine essential for proliferation (19–21). While a lot is known about Tregs and their role in tolerance and autoimmunity, very little has been published on the relationship between Tregs and RBC-specific autoimmunity. Recently, Mqadmi et al. (22) utilized a model in which AIHA is induced in mice by repeated transfusion of rat RBCs; in this model, Tregs were essential to mitigate autoimmunity. To build on these studies, we utilized a fully murine system (no xenoantigenic stimulus) and tested the requirement of Tregs in the establishment and maintenance of tolerance to a model antigen expressed on RBC precursors in the bone marrow and present throughout development and maturation of lymphocytes. In our model, sustained depletion of Tregs, or immunization with RBC-specific proteins following Treg depletion each failed to induce autoimmunity. Together, these data demonstrate that Tregs are a non-essential component of tolerance against RBC-specific antigens and suggest that other mechanisms are involved.

## Materials and Methods

### Mice

C57Bl/6 (B6) mice were purchased from NCI. B6.HOD (HOD) transgenic mice were generated as previously described and bred in the BloodworksNW vivarium (15, 23). Mice were maintained on standard rodent chow and water in a light- and temperature-controlled environment. B6 and HOD mice were used at 8–12 weeks of age and all procedures were performed according to protocols approved by the Bloodworks Northwest Institution Animal Care and Use Committee (IACUC).

### Treatment of Mice

B6 and HOD recipient mice were treated weekly with an intraperitoneal (i.p.) injection of 300 μg of anti-CD25 (BioXcell, clone PC-61) or anti-HRPN Rat IgG1 isotype control (BioXcell). In some experiments, recipient mice were immunized subcutaneously with 100 μg of ovalbumin (Sigma) emulsified in CFA (Difco Labs). Sera were collected 14 days post-treatment.

### MHCII Tetramer-Based Enrichment of Antigen-Specific Endogenous CD4+ T Cells

Leukocytes were harvested from spleen and lymph nodes, stained with pooled tetramer reagents, positively enriched, and stained with antibodies against cell surface markers as previously described (17). PE-conjugated tetramers utilized were: OVA329-337 (AAHAEINEA), OVA328-337 (HAAHAEINEA), and OVA325-335 (QAVHAAHAEIN). In some experiments, enriched leukocytes were stained with antibodies against CD25 (clone PC-61 or 7D4), GITR, and PD1 (eBioscience). Intracellular FoxP3 staining was performed as suggested by the manufacturer (eBioscience). To assess for viability, cells were stained with AmCyan viability dye (eBioscience).

### Antibody Detection

Flow crossmatch, direct antiglobulin test (DAT), and determination of anti-OVA antibodies (by ELISA) were performed as previously described (24, 25). To assess for HOD antigen expression, RBCs were stained with monoclonal antibodies specific for HEL (4B7) and Duffy (MIMA-29), as previously described (15, 17).

### Statistics

Significance was determined with a Student’s *T*-test for experiments with two groups or one-way ANOVA with a Bonferroni post-test for experiments with three or more groups. Significance was set at *p* < 0.05.

## Results

### Endogenous Autoreactive CD4+ T Cells in HOD Mice Express Regulatory Markers

**Figure 1:**
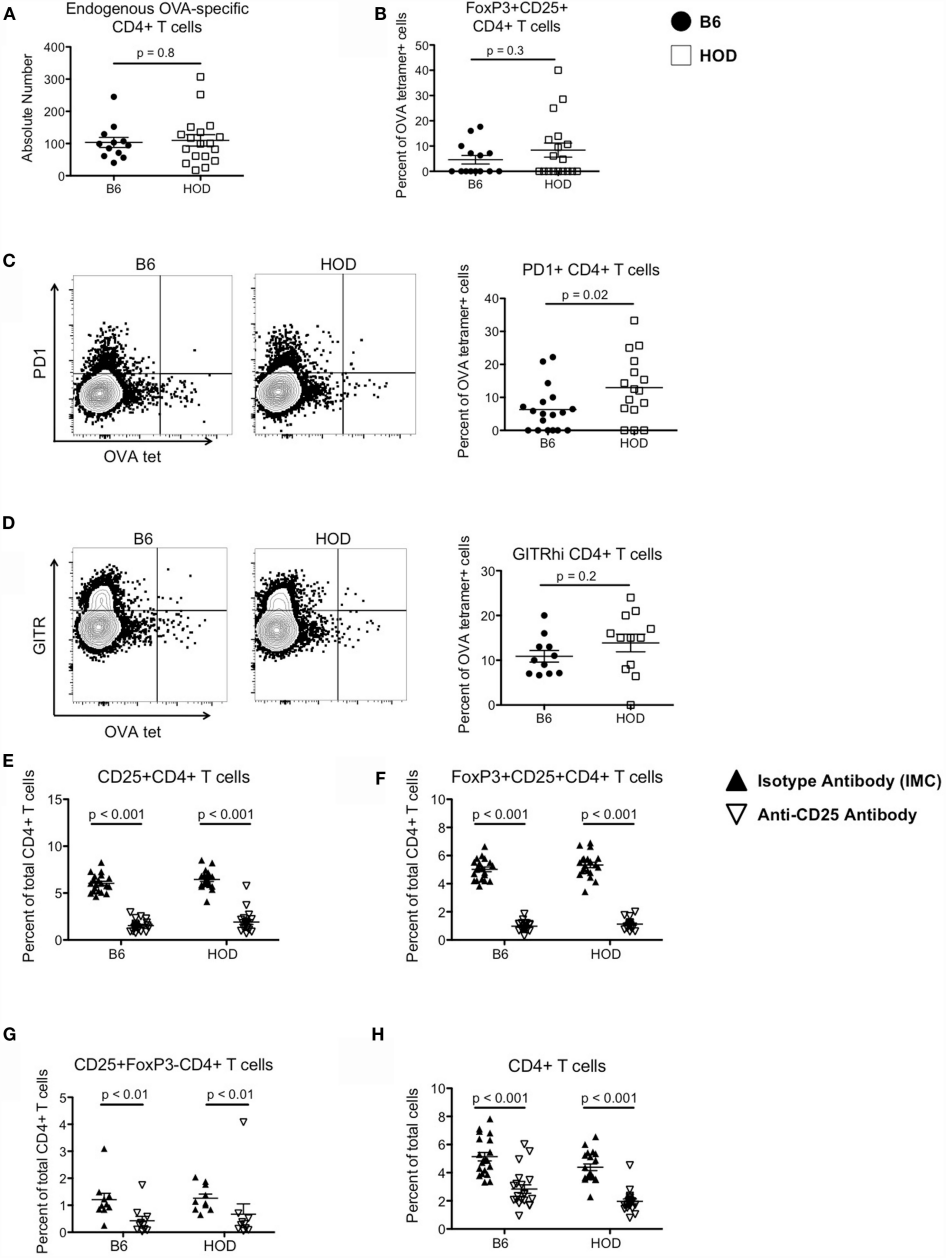
**Autoreactive OVA-specific CD4+ T cells in HOD mice express elevated levels of regulatory markers and can be depleted with anti-CD25 monoclonal antibodies**. **(A)** Endogenous numbers of OVA-specific CD4+ T cells in HOD and control B6 mice were determined by MHCII tetramer-based enrichment using pooled tetramers OVA329-337, OVA328-337, and OVA325-335. Data presented are gated on viable cells with phenotype CD4+CD3+CD8−CD19−CD11c−F4/80−CD11b−. **(B–D)** Enriched tetramer-binding T cells were evaluated for expression of regulatory T cell markers by staining for **(B)** surface CD25 (PC-61) and intracellular transcription factor FoxP3, **(C)** PD1, and **(D)** GITR. **(E–G)** To deplete CD4+CD25+FoxP3+ regulatory T cells, HOD and B6 mice were treated weekly with intraperitoneal doses of 300 μg of anti-CD25 monoclonal antibody (clone PC-61) or anti-HRPN Rat IgG1 isotype-matched control (IMC). Efficiency of depletion was evaluated 3 days post-treatment and frequencies of **(E)** CD25+CD4+ T cells, **(F)** FoxP3+CD25+CD4+ T cells, **(G)** CD25+FoxP3−CD4+ T cells, and **(H)** total CD4+ T cells were determined. Data shown are the cumulative results from three independent experiments with at least three mice per group.

To test the hypothesis that Tregs are required for prevention of RBC-specific autoantibodies, we utilized the HOD mouse model; as previously described, the RBC-restricted HOD antigen contains a triple fusion protein consisting of HEL, OVA, and human blood group molecule, Duffy (15). In our previous studies, we utilized MHCII tetramer reagents to detect RBC-specific T cells, specifically OVA-reactive CD4+ T cells (17); since our initial report, we have broadened our antigen coverage through implementation of an additional MHCII tetramer reagent. Thus, the current studies utilize pooled tetramer reagents for peptides OVA329-337 (AAHAEINEA), OVA328-337 (HAAHAEINEA), and OVA325-335 (QAVHAAHAEIN). To compare the number of endogenous CD4+ T cells that recognize OVA within both HOD and control B6 mice, we performed MHCII tetramer-based enrichments with pooled tetramer reagents. Enriched leukocytes were subsequently stained with antibodies against surface antigens CD3, CD4, CD8, CD19, CD11c, F4/80, and CD11b to delineate T cell populations. CD4+ T cell populations, defined as CD4+CD3+CD8−CD19−CD11c−F4/80−CD11b− were subsequently assessed for binding to OVA tetramers. To determine background staining for pooled OVA tetramers, CD4+ T cells that were not enriched were assessed for tetramer staining (Figure S1 in Supplementary Material). Evaluating the enriched CD4+ T cell population, as previously reported, B6 and HOD mice have similar absolute numbers of endogenous OVA-reactive CD4+ T cells (Figure 1A); however, pooling 3 MHCII tetramer reagents resulted in higher overall numbers (mean B6: 103; HOD: 109) than previously reported with only two tetramers (mean B6: 40; HOD: 56), suggesting that additional OVA-reactive CD4+ T cells can now be identified (17).

We previously observed that autoreactive endogenous OVA-specific CD4+ T cells in HOD mice do not respond to their cognate antigen and are non-functional; thus, it is plausible that these cells are Tregs or are under regulatory control exerted by Tregs. To test whether these OVA-reactive CD4+ T cells are Tregs, cells were co-stained for CD25 and FoxP3. Compared to control B6, HOD mice had a slightly higher, but not statistically significant, percentage of CD25+FoxP3+CD4+ T cells, a phenotype consistent with Tregs (Figure 1B). Moreover, the frequency of OVA-reactive CD4+ T cells that also expressed PD1 was significantly higher in HOD mice when compared to control B6 (Figure 1C). Representative dot plots of PD1 expression within OVA-tetramer reactive CD4+ T cells for both B6 and HOD are shown in Figure 1C left and middle, respectively. The frequency of PD1+OVA-reactive CD4+ T cells from all experimental mice is shown in graph form Figure 1C right. Additional analysis revealed that HOD mice had an elevated, but not significant, frequency of GITRhi+OVA-reactive CD4+ T cells in HOD mice (Figure 1D). Representative dot plots for B6 and HOD (Figure 1D left and middle) and frequencies from all experimental mice (Figure 1D right) are shown. Given that co-expression of CD25 and FoxP3, and both PD1 and GITR have all been associated with regulatory function of regulatory T cells (26, 27), these data suggest that a subset of autoreactive endogenous OVA-reactive CD4+ T cells in HOD mice express a phenotype that is consistent with Tregs.

### Depletion of Tregs Does Not Lead to Autoantibody Generation

To test whether Tregs are required to prevent RBC-specific autoantibodies, B6 and HOD mice were treated with anti-CD25 (clone PC-61), a regimen previously shown to deplete Tregs (26, 28). Control cohorts received Rat IgG1 isotype-matched control (IMC). Treg depletion was evaluated 3 days post-treatment by staining with anti-CD25 clone 7D4, an antibody with specificity for an epitope distinct from clone PC-61 (29). Anti-CD25, but not IMC, led to significant reduction in the percentage of CD25+CD4+ T cells and FoxP3+CD25+CD4+ Tregs, with similar frequencies detectable in both HOD and B6 mice (Figures 1E,F). Given that anti-CD25 monoclonal antibody may also affect other cell types that express CD25, total CD25+FoxP3−CD4+ effector T cells were also analyzed (30). Treatment with anti-CD25, but not IMC, led to a significant reduction of effector T cells in both HOD and control B6 mice (Figure 1G). Finally, there was a significant reduction in total CD4+ cells upon anti-CD25 treatment (Figure 1H).

**Figure 2:**
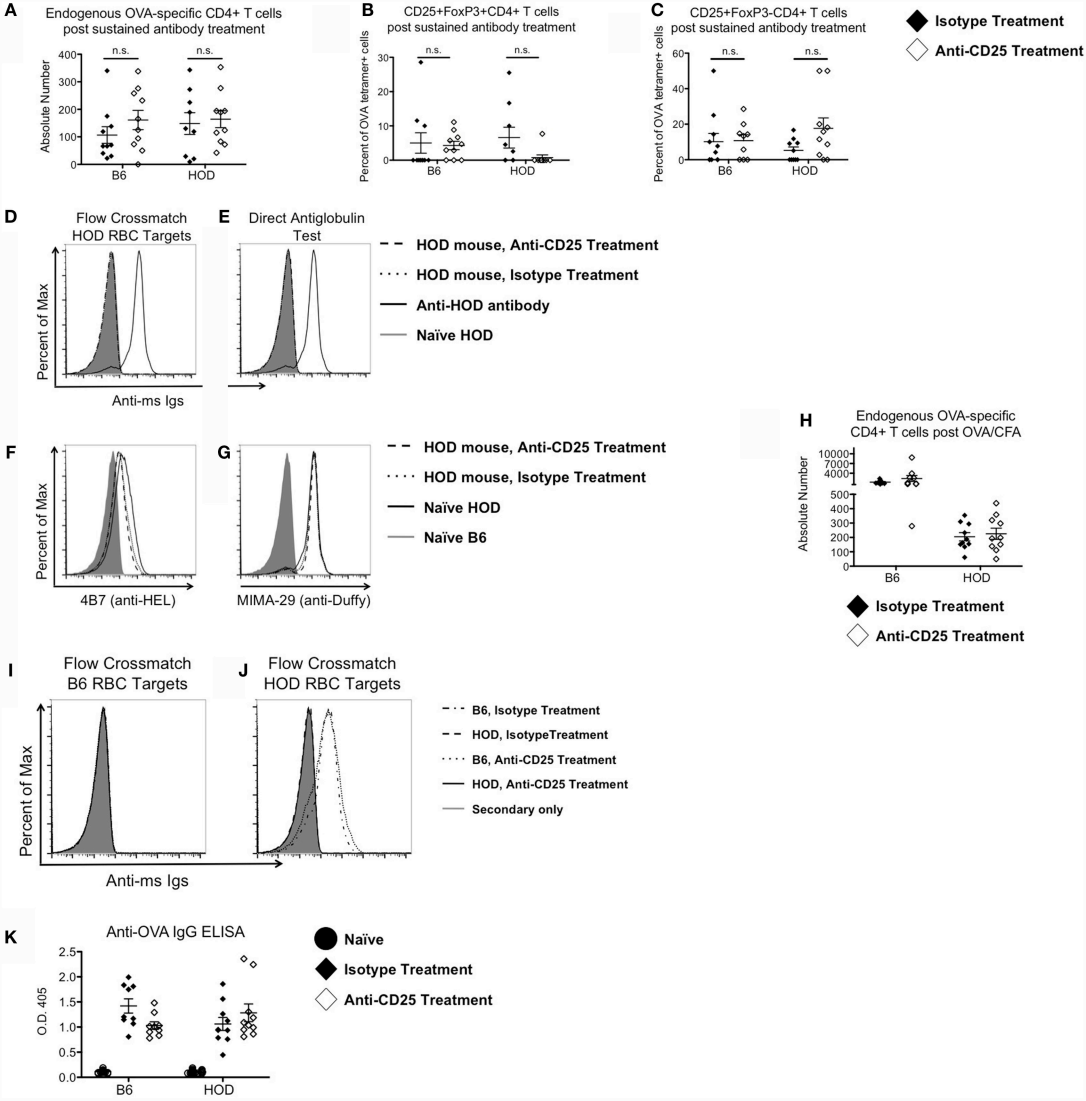
**Tregs are not required to prevent RBC-specific autoimmunity**. Control B6 and HOD mice were treated weekly for 4 weeks with intraperitoneal injections of 300 μg of anti-CD25 monoclonal antibody (PC-61) or anti-HRPN isotype-matched control (IMC). At the conclusion of treatment, **(A)** absolute numbers of endogenous OVA-reactive T cells were quantified, and the percentage of **(B)** CD25+FoxP3+CD4+ regulatory T cells and **(C)** CD25+FoxP3−CD4+ effector T cells were evaluated. **(D)** To evaluate autoantibody production, experimental serum was utilized for flow crossmatch analysis by incubation of serum with HOD target RBCs, followed by staining with an anti-mouse Ig secondary conjugated to APC (mIg APC). **(E)** To assess whether RBCs from experimental mice had autoantibodies bound to them, RBCs were stained with mIg APC. **(F,G)** To test for antigen modulation, experimental RBCs were assessed for expression of HOD antigen proteins by staining for **(F)** HEL with monoclonal antibody 4B7 and **(G)** Duffy with monoclonal antibody MIMA-29. In some experiments, experimental mice received a 100 μg OVA/CFA immunization at the midpoint of treatment with anti-CD25 or IMC. **(H)** Expansion of OVA-binding T cells was evaluated by MHCII tetramer-based enrichment 2 weeks post-immunization. Data presented are gated on CD4+CD3+CD8−CD19−CD11c−F4/80−CD11b− cells. **(I,J)** Autoantibody production was assessed by flow crossmatch whereby serum from experimental mice was incubated with **(I)** control B6 and **(J)** HOD RBC targets followed by mIg APC. **(K)** Production of OVA-specific antibodies by experimental mice was evaluated by ELISA.

To evaluate whether sustained depletion of Tregs led to anti-RBC autoantibodies, mice were maintained on the anti-CD25 or IMC regimen for 4 weeks, after which endogenous numbers of OVA-reactive CD4+ T cells were enumerated and assessed for co-expression of CD25 and FoxP3. Prolonged treatment with monoclonal antibodies did not significantly affect the absolute numbers of detectable OVA-reactive CD4+ T cells in HOD or control B6 mice (Figure 2A). However, within the OVA-reactive CD4+ T cell population, the percentage of CD25+FoxP3+CD4+ Tregs was decreased in HOD animals treated with anti-CD25 when compared to IMC (Figure 2B). Likewise, prolonged treatment with anti-CD25 or IMC did not significantly alter the percentage of CD25+FoxP3−CD4+OVA-reactive effector T cells (Figure 2C). Humoral responses post monoclonal antibody treatment were also evaluated. Detection of RBC-specific antibodies was assessed by flow crossmatch. Briefly, sera from experimental mice were incubated with HOD or control B6 RBCs followed by staining with anti-mouse immunoglobulin secondary conjugated to APC (mIg APC). Neither control B6 nor HOD animals that received anti-CD25 or IMC made detectable anti-HOD antibodies (data not shown and Figure 2D). To assess whether autoantibodies were secreted but were binding directly to RBCs, RBCs from experimental mice were incubated with mIg APC, an assay known as a DAT. Neither B6 nor HOD mice had detectable antibodies bound to their RBCs (data not shown and Figure 2E). An alternate hypothesis is that HOD antibodies were produced in HOD mice, binding to HOD antigen, but were mediating antigen modulation. To test this hypothesis, RBCs from experimental HOD mice were stained with 4B7 (an anti-HEL monoclonal antibody) or MIMA-29 (an anti-Duffy monoclonal antibody) followed by mIg APC (17, 31). Naïve, anti-CD25-treated, and IMC-treated HOD animals had similar expression patterns of detectable HEL and Duffy on their RBC surface, demonstrating that antigen modulation had not occurred (Figures 2F,G). Taken together, a sustained absence of Tregs did not lead to autoantibody production in HOD or control B6 mice. Furthermore, these data suggest that Tregs are not required to prevent autoreactive endogenous OVA-reactive CD4+ T cells (within HOD mice) from activating.

### Treg Depletion in Conjunction with Immunization Does Not Elicit Autoantibodies in HOD Mice

Development of autoimmunity is postulated to require multiple hits, with each “hit” weakening tolerance mechanisms (32). To test whether immunization in the absence of Tregs leads to autoantibodies, B6 and HOD mice received anti-CD25 or IMC for 4 weeks. At the midpoint of treatment, experimental mice received an OVA/CFA immunization. Mice were sacrificed 2 weeks post-immunization and total endogenous OVA-reactive T cells were enumerated by MHCII tetramer-based enrichment. Significant expansion of endogenous OVA-reactive CD4+ T cells was observed in control B6 mice immunized with OVA/CFA (mean IMC: 1273; anti-CD25: 2379) (Figure 2H); however, there was no significant expansion in OVA/CFA immunized HOD animals, regardless of IMC or anti-CD25 treatment. To assess for production of HOD-specific antibodies, sera were analyzed by flow crossmatch against B6 and HOD RBC targets (Figures 2I,J). Sera from B6 but not HOD mice (both anti-CD25 and IMC-treated groups) made antibodies to the HOD antigen. Given that an OVA/CFA injection into HOD mice failed to elicit OVA-specific T cell expansion and to promote HOD-specific antibody generation, we performed an OVA-specific ELISA to test the hypothesis that the OVA/CFA immunization was successful in HOD mice. By design, the HOD antigen only contains the C-terminal half of OVA (15). Thus, an OVA/CFA immunization with the whole OVA protein is postulated to elicit antibodies to both the C- and N-terminal regions of the protein. As such, an ELISA containing the whole OVA protein was utilized to evaluate the effectiveness of the OVA/CFA immunization. OVA-specific IgG antibodies were detectable in both B6 and HOD mice that received an OVA/CFA immunization, regardless of antibody treatment (Figure 2K), suggesting that HOD mice made OVA-specific antibodies to the N-terminal portion of OVA, which is not contained within the HOD antigen. Together, these data demonstrate that immunization in combination with Treg depletion is not sufficient to break down tolerance and lead to autoantibodies in HOD animals. It further suggests that neither RBC-specific Tregs nor immunosuppressive effects by Tregs are required to prevent anti-RBC autoantibodies.

## Discussion

Failure of tolerance mechanisms to RBC antigens can lead to development of pathogenic autoantibodies, which can mediate destruction of RBCs and result in AIHA, a severe and sometimes fatal disease. Using the HOD mouse model, we have previously reported that tolerance to RBC self-antigens is incomplete in the B cell compartment; however, stringent T cell tolerance prevents RBC-specific autoreactive B cell activation and subsequent autoantibody production. One potential mechanism of T cell tolerance employed to prevent autoimmunity is through immunosuppression by Tregs (33, 34). As evidence of their immunosuppressive capabilities, in both human trials and murine models, transient depletion or functional inactivation of Tregs has been documented to be an effective strategy to enhance anti-tumor responses and treat chronic infections (35, 36). Given their potency, we hypothesized that Tregs were required for maintenance of tolerance to RBC-specific autoantigens. However, data presented herein suggest that Tregs are not essential to maintain tolerance to RBC autoantigens.

RBC-specific autoreactive T cells are detectable in HOD mice. However, these autoreactive CD4+ T cells are non-functional and co-express CD25 and FoxP3, a phenotype consistent with Tregs. Additional phenotypic characterization revealed that these autoreactive T cells express elevated levels of PD1 and GITR, surface markers associated both with regulation and exhaustion. Given that RBCs have a life span of ~50 days in the mouse (or ~120 days in humans), and are continually renewed by the bone marrow, autoreactive T cells undergo chronic exposure to large amounts of RBC-derived antigens. Accordingly, T cells in our model express both regulatory and exhaustion cellular markers, a phenotype associated with T cells exposed to chronic cognate autoantigen. Functionally, T cells that encounter persistent antigen may become anergic, which is characterized by inhibition of proliferation and effector functions. There are several categories of anergy, some of which can be reversed through cytokine exposure, providing costimulation, or removing autoreactive T cells into an environment that lacks their autoantigen (20). Aspects of anergy (e.g., lack of proliferation) are found in the Treg compartment as well, making it difficult to discern between the anergic states (10). However, phenotypic analysis suggested that HOD-specific autoreactive T cells could be Tregs.

Canonically, Tregs express high levels of surface CD25, a receptor that binds to proliferative cytokine IL-2. Through CD25 expression, Tregs can mediate immune suppression through IL-2 scavenging, thereby depriving other cells (e.g., T cells) of this proliferation-inducing cytokine and successfully dampening subsequent immune responses (37). Targeting CD25 with monoclonal antibodies has been an effective way to inhibit Treg function, which has provided insight into the mechanistic understanding of how Tregs modulate immune responses. Although the field remains divided upon the mechanism of action of anti-CD25 antibodies (29, 38), data with our model (and others) suggest that monoclonal anti-CD25 treatment induced significant deletion of the CD25-expressing CD4+ T cells, both CD25+FoxP3+CD4+ Tregs and CD25+FoxP3−CD4+ effector T cells (30). While complete depletion of CD25+FoxP3+CD4+ T cells is not achieved with this approach, over 75% of total Tregs became undetectable post anti-CD25 treatment; furthermore, OVA-specific Tregs, but not overall numbers, were reduced in anti-CD25 treated HOD mice. This treatment regimen has been documented to effectively result in inhibition of Treg function and is sufficient to promote autoantibody synthesis. However, upon treatment of HOD mice with anti-CD25 antibodies for 4 continuous weeks, no autoantibodies were detected. Thus, these data suggest that prevention of RBC-specific autoantibody generation is not dependent upon the regulatory function of Tregs.

Initiation of autoimmune disease has been postulated to occur in response to or concurrent with infection or excessive immune activation in otherwise immunocompetent individuals. As such, a complex microbial history may play a role in the development of autoimmunity. Indeed, there is a higher frequency of autoimmunity in the elderly, a population that has potentially encountered more pathogens, compared to younger age groups (39). Upon each pathogen encounter, the immune response may give rise to new cross-reactive lymphocytes, such that the lymphocyte can react to both foreign antigen and self-antigen. Furthermore, inflammation elicited in response to pathogens may induce bystander activation of autoreactive cells. To test whether inflammation, in combination with an absence of immune regulation by Tregs, and exposure to cognate antigen could lead to autoantibodies, HOD mice were treated with anti-CD25 and then immunized with OVA/CFA. CFA elicits a strong immune response, with copious TH1 cytokine production, including IL-2 (40). In control mice, robust proliferation of OVA-specific T cells was detected, whereas there was no significant expansion in HOD mice. Thus, even with a strong immune stimulus and lack of immunosuppressive cytokines, autoreactive HOD CD4+ T cells were still not capable of providing adequate help to B cells thereby adding support to our hypothesis that Tregs are dispensable for the prevention of RBC-specific autoreactive antibodies.

Anti-CD25 monoclonal antibody treatment targets not only CD25+FoxP3+ Tregs but also CD25+ effector T cells, which are critical to providing B cell help to elicit antibodies. Thus, it is plausible that anti-CD25 treatment would impact antibody production. However, in both B6 and HOD mice that received sustained antibody treatment in conjunction with an OVA/CFA immunization, anti-OVA antibody titers were similar between anti-CD25 and IMC groups, demonstrating that B cell received adequate help (Figure 2J). These data, in combination with Figure 2C demonstrating that OVA-reactive effector cells are not significantly affected by anti-CD25 treatment, suggest that despite significant reductions of CD25+ T cells, there are enough remaining effector cells to provide B cell help for antibody production. Alternatively, effector T cells may stimulate anti-OVA antibody production in a CD25-independent fashion (41). Regardless of mechanism, while anti-CD25 treatment may affect cells that transiently express CD25, monoclonal antibody treatment does not prevent antibody production in this model. Thus, given that anti-HOD antibodies are not detected in HOD animals treated with anti-CD25 and OVA/CFA, these data, in aggregate, suggest that other regulatory mechanisms besides Tregs play an important role in maintaining tolerance to RBC antigens.

In summary, our findings suggest that Tregs are not required for the prevention of RBC autoimmunity; however, we cannot rule out that Tregs may be involved, as multiple redundant tolerance pathways may be utilized to maintain tolerance to RBC antigens. Indeed, establishment of T cell tolerance can be achieved through deletion, anergy, and regulation; thus, it is possible that multiple pathways are utilized for a specific antigen-reactive T cell population. These data suggest that, unlike most tissue-specific antigens, Tregs are not required for preventing the development of RBC-specific autoimmunity. However, the specific tolerance mechanisms employed against RBC-specific antigens is stringent and cannot be compromised by the absence of Tregs, even in the presence of strong immune activation. Taken together, these data suggest that other tolerance pathways are likely involved.

## Author Contributions

AR and KH designed and performed research, analyzed data, and wrote paper. XW and LK performed research and analyzed data. HH evaluated data and corrected the paper. All authors read and edited the manuscript.

## Conflict of Interest Statement

The authors declare that the research was conducted in the absence of any commercial or financial relationships that could be construed as a potential conflict of interest.

## Supplementary Material

Figure S1 **Gating strategy for OVA-tetramer-binding CD4+ T cells**. Unenriched leukocytes were utilized to determine the background staining for OVA-specific MHCII tetramers. Leukocytes were gated on CD4+CD3+CD8−CD19−CD11c−F4/80−CD11b− and evaluated for OVA-tetramer binding and positive staining gate was drawn.

## Abbreviations

AIHA, autoimmune hemolytic anemia; CFA, complete Freund’s adjuvant; HEL, hen egg lysozyme; HOD, transgenic mouse that expresses RBC-specific transgene HEL-OVA-Duffy; IMC, isotype-matched control; mIg-APC, anti-mouse Ig conjugated to APC; OVA, ovalbumin; RBC, red blood cell; Tregs, regulatory T cells.

## References

[1] Gehrs BC Friedberg RC. Autoimmune hemolytic anemia. Am J Hematol (2002) 69(4):258–71. 10.1002/ajh.10062 11921020

[2] Dierickx D Kentos A Delannoy A. The role of rituximab in adults with warm antibody autoimmune hemolytic anemia. Blood (2015) 125:3223–9. 10.1182/blood-2015-01-588392 25827833

[3] Lechner K Jager U. How I treat autoimmune hemolytic anemias in adults. Blood (2010) 116:1831–8. 10.1182/blood-2010-03-259325 20548093

[4] Vos GH Petz LD Hugh Fudenberg H Specificity and immunoglobulin characteristics of autoantibodies in acquired hemolytic anemia. J Immunol (1971) 106:1172–6. 4995749

[5] Naik R. Warm autoimmune hemolytic anemia. Hematol Oncol Clin North Am (2015) 29:445–53. 10.1016/j.hoc.2015.01.001 26043384

[6] Kyewski B Klein L. A central role for central tolerance. Annu Rev Immunol (2006) 24:571–606. 10.1146/annurev.immunol.23.021704.115601 16551260

[7] Tussiwand R Bosco N Ceredig R Rolink AG. Tolerance checkpoints in B-cell development: Johnny B good. Eur J Immunol (2009) 39:2317–24. 10.1002/eji.200939633 19714572

[8] Xing Y Hogquist KA T-cell tolerance: central and peripheral. Cold Spring Harb Perspect Biol (2012) 4(6):a006957 10.1101/cshperspect.a006957 22661634 PMC3367546

[9] Nemazee D. Receptor editing in lymphocyte development and central tolerance. Nat Rev Immunol (2006) 6:728–40. 10.1038/nri1939 16998507

[10] Macian F Im SH Garcia-Cozar FJ Rao A. T-cell anergy. Curr Opin Immunol (2004) 16:209–16. 10.1016/j.coi.2004.01.013 15023415

[11] Yamamoto T Hattori M Yoshida T. Induction of T-cell activation or anergy determined by the combination of intensity and duration of T-cell receptor stimulation, and sequential induction in an individual cell. Immunology (2007) 121:383–91. 10.1111/j.1365-2567.2007.02586.x 17376194 PMC2265954

[12] Ashour HM Seif TM The role of B cells in the induction of peripheral T cell tolerance. J Leukoc Biol (2007) 82:1033–9. 10.1189/jlb.0507310 17656652

[13] Lewis KL Reizis B. Dendritic cells: arbiters of immunity and immunological tolerance. Cold Spring Harb Perspect Biol (2012) 4(8):a007401. 10.1101/cshperspect.a007401 22855722 PMC3405856

[14] Lerner A Jeremias P Matthias T The world incidence and prevalence of autoimmune diseases is increasing. Int J Celiac Dis (2015) 3:151–5. 10.12691/ijcd-3-4-8

[15] Desmarets M Cadwell CM Peterson KR Neades R Zimring JC. Minor histocompatibility antigens on transfused leukoreduced units of red blood cells induce bone marrow transplant rejection in a mouse model. Blood (2009) 114:2315–22. 10.1182/blood-2009-04-214387 19525479 PMC2745850

[16] Liu J Guo X Mohandas N Chasis JA An X. Membrane remodeling during reticulocyte maturation. Blood (2010) 115:2021–7. 10.1182/blood-2009-08-241182 20038785 PMC2837329

[17] Hudson KE Hendrickson JE Cadwell CM Iwakoshi NN Zimring JC. Partial tolerance of autoreactive B and T cells to erythrocyte-specific self-antigens in mice. Haematologica (2012) 97:1836–44. 10.3324/haematol.2012.065144 22733018 PMC3590090

[18] Sakaguchi S. Naturally arising CD4+ regulatory T cells for immunologic self-tolerance and negative control of immune responses. Annu Rev Immunol (2004) 22:531–62. 10.1146/annurev.immunol.21.120601.141122 15032588

[19] Barron L Dooms H Hoyer KK Kuswanto W Hofmann J O’Gorman WE Cutting edge: mechanisms of IL-2−dependent maintenance of functional regulatory T cells. J Immunol (2010) 185:6426–30. 10.4049/jimmunol.0903940 21037099 PMC3059533

[20] Schwartz RH. T cell anergy. Annu Rev Immunol (2003) 21:305–34. 10.1146/annurev.immunol.21.120601.141110 12471050

[21] Schietinger A Greenberg PD. Tolerance and exhaustion: defining mechanisms of T cell dysfunction. Trends Immunol (2014) 35:51–60. 10.1016/j.it.2013.10.001 24210163 PMC3946600

[22] Mqadmi A Zheng X Yazdanbakhsh K. CD4+CD25+ regulatory T cells control induction of autoimmune hemolytic anemia. Blood (2005) 105:3746–8. 10.1182/blood-2004-12-4692 15637139 PMC1895013

[23] Richards AL Hendrickson JE Zimring JC Hudson KE. Erythrophagocytosis by plasmacytoid dendritic cells and monocytes is enhanced during inflammation. Transfusion (2016) 56:905–16. 10.1111/trf.13497 26843479

[24] Hudson KE Lin E Hendrickson JE Lukacher AE Zimring JC. Regulation of primary alloantibody response through antecedent exposure to a microbial T-cell epitope. Blood (2010) 115:3989–96. 10.1182/blood-2009-08-238568 20086249 PMC2869558

[25] Zimring JC Hair GA Chadwick TE Deshpande SS Anderson KM Hillyer CD Nonhemolytic antibody-induced loss of erythrocyte surface antigen. Blood (2005) 106:1105–12. 10.1182/blood-2005-03-1040 15831698

[26] Nocentini G Alunno A Petrillo M Bistoni O Bartoloni E Caterbi S Expansion of regulatory GITR+CD25low/-CD4+ T cells in systemic lupus erythematosus patients. Arthritis Res Ther (2014) 16:444. 10.1186/s13075-014-0444-x 25256257 PMC4209023

[27] Martin-Orozco N Wang YH Yagita H Dong C Cutting edge: programed death (PD) ligand-1/PD-1 interaction is required for CD8+ T cell tolerance to tissue antigens. J Immunol (2006) 177:8291–5. 10.4049/jimmunol.177.12.8291 17142723

[28] Boehm F Martin M Kesselring R Schiechl G Giessler EK Schlitt HJ Deletion of Foxp3+ regulatory T cells in genetically targeted mice supports development of intestinal inflammation. BMC Gastroenterol (2012) 12:97. 10.1186/1471-230X-12-97 22849659 PMC3449180

[29] Setiady YY Coccia JA Park PU. In vivo depletion of CD4+FOXP3+ Treg cells by the PC61 anti-CD25 monoclonal antibody is mediated by FcgammaRIII+ phagocytes. Eur J Immunol (2010) 40:780–6. 10.1002/eji.200939613 20039297

[30] Couper KN Lanthier PA Perona-Wright G Kummer LW Chen W Smiley SS Anti-CD25 antibody mediated depletion of effector T cell populations enhances susceptibility of mice to acute, but not chronic, *Toxoplasma gondii* infection. J Immunol (2009) 182:3985–94. 10.4049/jimmunol.0803053 19299696 PMC3942880

[31] Zimring JC Cadwell CM Spitalnik SL. Antigen loss from antibody-coated red blood cells. Transfus Med Rev (2009) 23:189–204. 10.1016/j.tmrv.2009.03.002 19539874

[32] von Herrath MG Fujinami RS Whitton JL. Microorganisms and autoimmunity: making the barren field fertile? Nat Rev Micro (2003) 1:151–7. 10.1038/nrmicro754 15035044

[33] Kim JM Rasmussen JP Rudensky AY. Regulatory T cells prevent catastrophic autoimmunity throughout the lifespan of mice. Nat Immunol (2007) 8:191–7. 10.1038/ni1428 17136045

[34] Stephens LA Gray D Anderton SM. CD4+CD25+ regulatory T cells limit the risk of autoimmune disease arising from T cell receptor crossreactivity. Proc Natl Acad Sci U S A (2005) 102:17418–23. 10.1073/pnas.0507454102 16287973 PMC1297676

[35] Peng G Li S Wu W Sun Z Chen Y Chen Z Circulating CD4(+) CD25(+) regulatory T cells correlate with chronic hepatitis B infection. Immunology (2008) 123:57–65. 10.1111/j.1365-2567.2007.02691.x 17764450 PMC2433275

[36] Colombo MP Piconese S Regulatory T-cell inhibition versus depletion: the right choice in cancer immunotherapy. Nat Rev Cancer (2007) 7:880–7. 10.1038/nrc2250 17957190

[37] Shevach EM. Mechanisms of Foxp3+ T regulatory cell-mediated suppression. Immunity (2009) 30:636–45. 10.1016/j.immuni.2009.04.010 19464986

[38] Kohm AP McMahon JS Podojil JR Begolka WS DeGutes M Kasprowicz DJ Cutting edge: anti-CD25 monoclonal antibody injection results in the functional inactivation, not depletion, of CD4+CD25+ T regulatory cells. J Immunol (2006) 176:3301–5. 10.4049/jimmunol.176.6.3301 16517695

[39] Vadasz Z Haj T Kessel A Toubi E. Age-related autoimmunity. BMC Med (2013) 11:94. 10.1186/1741-7015-11-94 23556986 PMC3616810

[40] Billiau A Matthys P Modes of action of Freund’s adjuvants in experimental models of autoimmune diseases. J Leukoc Biol (2001) 70:849–60. 11739546

[41] Colombetti S Basso V Mueller DL Mondino A. Prolonged TCR/CD28 engagement drives IL-2-independent T cell clonal expansion through signaling mediated by the mammalian target of rapamycin. J Immunol (2006) 176:2730–8. 10.4049/jimmunol.176.5.2730 16493028

