# Supplementary figures and images for: Regulatory T Cells Are Dispensable for Tolerance to RBC Antigens
Source: Front Immunol. 2016 Sep 19;7:348. doi: 10.3389/fimmu.2016.00348 (PMC5027202; doi:10.3389/fimmu.2016.00348)

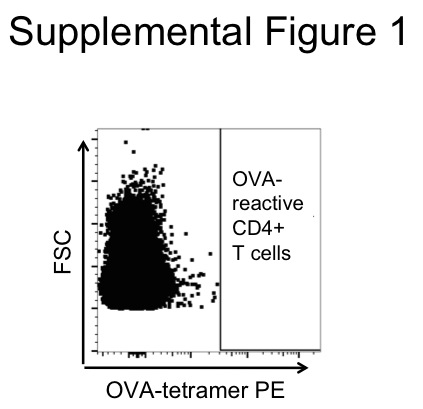

Supplement: Figure S1 — Gating strategy for OVA-tetramer-binding CD4+ T cells. Unenriched leukocytes were utilized to determine the background staining for OVA-specific MHCII tetramers. Leukocytes were gated on CD4+CD3+CD8−CD19−CD11c−F4/80−CD11b− and evaluated for OVA-tetramer binding and positive staining gate was drawn. [file image_1.jpeg]
